# Blockade of TRPM7 Alleviates Chondrocyte Apoptosis and Articular Cartilage Damage in the Adjuvant Arthritis Rat Model Through Regulation of the Indian Hedgehog Signaling Pathway

**DOI:** 10.3389/fphar.2021.655551

**Published:** 2021-04-13

**Authors:** Ganggang Ma, Yang Yang, Yong Chen, Xin Wei, Jie Ding, Ren-Peng Zhou, Wei Hu

**Affiliations:** ^1^ Department of Clinical Pharmacology, The Second Hospital of Anhui Medical University, Hefei, China; ^2^ The Key Laboratory of Anti-inflammatory and Immune Medicine, Anhui Medical University, Ministry of Education, Hefei, China

**Keywords:** arthritis, TRPM7, 2-APB, indian hedgehog, apoptosis

## Abstract

Articular cartilage damage with subsequent impairment of joint function is a common feature of articular diseases, in particular, rheumatoid arthritis and osteoarthritis. While articular cartilage injury mediated by chondrocyte apoptosis is a known major pathological feature of arthritis, the specific mechanisms remain unclear at present. Transient receptor potential melastatin-like seven channel (TRPM7) is reported to play an important regulatory role in apoptosis. This study focused on the effects of TRPM7 on arthritic chondrocyte injury and its underlying mechanisms of action. Sodium nitroprusside (SNP)-induced rat primary chondrocyte apoptosis and rat adjuvant arthritis (AA) were used as *in vitro* and *in vivo* models, respectively. Blockage of TRPM7 with 2-APB or specific siRNA resulted in increased chondrocyte viability and reduced toxicity of SNP. Moreover, treatment with 2-APB enhanced the Bcl-2/Bax ratio and reduced cleaved PARP and IL-6, MMP-13 and ADAMTS-5 expression in SNP-treated chondrocytes. Activation of Indian Hedgehog with purmorphamine reversed the protective effects of 2-APB on SNP-induced chondrocyte apoptosis. Blockage of TRPM7 with 2-APB relieved the clinical signs of AA in the rat model and reduced the arthritis score and paw swelling. Similar to findings in SNP-treated chondrocytes, 2-APB treatment increased the Bcl-2/Bax ratio and suppressed cleaved PARP, IL-6, MMP-13, ADAMTS-5, TRPM7, and Indian hedgehog expression in articular cartilage of AA rats. Our collective findings suggest that blockade of TRPM7 could effectively reduce chondrocyte apoptosis and articular cartilage damage in rats with adjuvant arthritis through regulation of the Indian Hedgehog signaling pathway.

## Introduction

Arthritis is an acute or chronic inflammation disease that could affect multiple joints (Harth and Nielson, 2019) and cause synovial swelling, monocyte infiltration, stiffness in the joints, pannus formation and destruction of articular cartilage (Tang, 2019). Metabolic abnormalities and crystal deposition of rheumatoid arthritis (RA) and degenerative changes of osteoarthritis could cause articular cartilage damage (Harth and Nielson, 2019). Cartilage damage in arthritis is mainly the proteolytic disorder of the extracellular matrix of cartilage cells. Members of the matrix metalloprotease and metalloprotease and "A disintegrin with thrombospondin motifs" families jointly answer for cartilage catabolism (Rowan et al.,2008). At present, the pathogenesis and mechanisms of articular cartilage damage is still unknown. Several genetic and environmental factors cause RA and chondrocyte apoptosis increase (Zhou et al., 2016). Inhibition of apoptosis has been shown to reduce bone damage and improve disease (Liu et al., 2018). Thus, the strategy aimed at the key factors and mechanisms of regulating chondrocyte apoptosis hold promise as potential treatments for arthritis.

TRPM7 is a nonselective membrane channel protein of the TRPM subfamily (Cabezas-Bratesco et al., 2015). At the same time, TRPM7 is also a *α*-kinase with less amino acid sequence semblance to general protein kinases (Zou et al., 2019). The gene of TRPM7 is located on chromosome 15q21.2, and each TRPM7 protein has six transmembrane fragments (S1-S6), S1-S4 fragments are sensors, and there are channels between S5 and S6 fragments (Zhang et al., 2018a; Zou et al., 2019). The channel portion of TRPM7 conducts influx of divalent cations, such as Ca^2+^, Mg^2+^, and Zn^2+^ (Oh and Chung, 2017). Ca^2+^ is involved in several physiological and pathological processes, and therefore, TRPM7-Mediated Ca^2+^ has attracted significant research attention. Earlier studies suggest that intracellular [Ca^2+^]_i_ participates in chondrocyte apoptosis in RA (Wu et al.,2019). TRPM7 plays significant roles in cell growth, metastasis, inflammation, fibrosis, and programmed cell death (Huang et al., 2018; Liu et al., 2019; Numata et al., 2019; Rios et al., 2020). In HEK293 cells, mouse hippocampal neurons and neonatal stroke models, TRPM7 overexpression promotes apoptosis while its inhibition has a converse effect (Chen et al., 2015). However, the issue of whether TRPM7 plays a role in apoptosis of articular chondrocytes remains to be established.

The Drosophila Hedgehog (HH) gene discovered in 1992 encodes a class of secreted signaling molecules and plays a critical role in many developmental processes in mammalian tissues (Ingham and McMahon, 2001; Taipale and Beachy, 2001). Indian Hedgehog (IHH) belonging to the HH family is a secreted protein produced by prehypertrophic chondrocytes (van den Brink, 2007; Lu et al., 2017). IHH plays significant roles in bone development and growth and differentiation of chondrocytes (Al-Azab et al., 2018). Abnormal activation of the IHH signal can trigger a variety of bone diseases, such as progressive bone dysplasia (Deng et al., 2018). Expression of IHH increases in the early stages of cartilage damage (Thompson et al., 2015), and the enhanced levels of associated genes, Glioma-associated one and smoothened could promote RA development through regulatory effects on MMP production (Al-Azab et al., 2018). One study demonstrated that TRPM7 silence could reduce the expression of IHH (Lu et al., 2017), while the relationship between TRPM7 and IHH and its roles in arthritis cartilage damage remains unclear. Here, we employed a variety of techniques, including MTT analysis, Lactate Dehydrogenase (LDH) assay, Hoechst 33,258, Rh123 and ROS staining, western blot, quantitative real-time PCR (qRT-PCR), flow cytometry, TRPM7 small interference (Si) RNA for transfecting chondrocytes, and radiological and immunohistochemical analyses, to determine the effects of TRPM7 and IHH in AA.

## Materials and Methods

### Separation of Chondrocytes and Cell Culture

Male Sprague-Dawley (SD) rats weighing 160–180 g were obtained from Jinan Pengyue Experimental Animal Breeding Co., Ltd., China. After adaptation for 7 days, SD rats were used to separation of chondrocytes. Under aseptic conditions, chondrocytes were extracted from the tibial platform of 52 male SD rats. The extracted knee cartilage was placed in culture dish, washed twice with PBS, cut into small pieces (<1 mm^3^) and then washed twice with PBS until colorless remained. Cartilage pieces were digested with 0.25% trypsin solution for 30 min, washed with PBS until colorless, digested with 0.2% type II collagenase for 3 h in culture dish in the incubator, and then centrifuged at 1,500 rpm to obtain chondrocytes. Chondrocytes were cultured in Dulbecco's modified eagle medium (DMEM, Hyclone, United States) containing 10% fetal bovine serum (FBS, Gibco, United States) in a culture flask in incubator at a CO_2_ concentration of 5% at 37°C, and used within the first three passages. Chondrocytes were seeded at a density of 5 × 10^3^ cells/well in 96-well plates to detect cell viability (Zhou et al., 2018a) and a density of 5 × 10^4^ cells/well in 24-well plates to detect cytotoxicity (Chang et al., 2006). Chondrocytes were seeded at a density of 5 × 10^5^ cells/well in 6-well plates to stain Hoechst33258 and Rh123 and a density of 2 × 10^6^ cells/well in 6-well plates for western blot, qRT-PCR and flow cytometry assay (Grossin et al., 2004; Sun et al., 2019). Toluidine blue and type II collagen staining were used to identify chondrocytes.

### MTT Assay

Cell viability was evaluated by the MTT. Chondrocytes were seeded at a density of 5 × 10^3^ cells/well in 96-well plates, cultured for 24 h, and then treated with SNP (0.125–2 mM) for 12 h. Chondrocytes were treated with SNP (0.5 mM) for 1.5–24 h, or treated with 2-APB (25–400 μM) for 12 h. Chondrocytes were combined the treatment of SNP (0.5 mM) and 2-APB (50–200 μM) for 12 h. Chondrocytes were treated with SNP (0.5 mM), 2-APB (100 μM) and PMA (4 μM) for 12 h. Then 10 μl of 5 mg/ml MTT (Beyotime, China) dissolved in PBS was added to each well of a 96-well plate. After that, cells were placed in the incubator at 37°C for 4 h. Subsequently, 150 μl dimethyl sulfoxide was added to dissolve formazan crystals. After a 10 min incubation period, absorbance values of living cells were read at 492 nm. Cell viability was expressed as a percentage relative to the control group (control %).

### Lactate Dehydrogenase (LDH) Assay

Cytotoxicity was measured by using the LDH assay. Chondrocytes were seeded at a density of 5 × 10^4^ cells/well in 24-well plates, cultured for 24 h, and then treated with SNP (0.5 mM) and 2-APB (100 μM) for 12 h. Firstly, the medium was aspirated (50 μl per well) from 24-well plates into 96-well plates for detection of LDH release and chondrocytes lysed with Triton X-100 for 30 min to obtain maximum LDH release. Then medium was aspirated (50 μl per well) from 24-well containing Triton X-100 into 96-well plates and 50 μl reagent added from the Cytotoxicity Detection Kit (Roche Diagnostics) to each well. The 96-well plate was preincubated for 30 min in the dark, and absorbance detected at 620 and 492 nm using a spectrometer (Thermo Fisher Scientific, United States). LDH release was determined based on subtracting absorbance at 620 nm from that at 492 nm. LDH from lysed cells using Triton X was the maximum LDH release and used as a standardizer to obtain the relative release of LDH. Relative LDH release = LDH release/maximum LDH release.

### Hoechst33258 and Rh123 Staining

Chromatin of the nucleus was detected by Hoechst33258 staining and mitochondrial membrane potential was detected by Rh123 staining. Chondrocytes were seeded at a density of 5 × 10^5^ cells/well in 6-well plates, cultured for 24 h, and then treated with SNP (0.5 mM) and 2-APB (100 μM) for 12 h. After that, Hoechst33258 was directly added to culture plates, reacted in the dark for 30 min, and washed twice in PBS. Cells were examined under a fluorescence microscope. Following treatment, chondrocytes were washed twice in PBS, treated with Rh123 (1:1,000), reacted in the dark for 30 min, and observed under a fluorescence microscope.

### Flow Cytometry Assay

For measurement of apoptosis, cells were seeded at a density of 2 × 10^6^ cells/well in 6-well plates and cultured for 24 h, then treated with SNP (0.5 mM) and 2-APB (100 μM) for 12 h. Collected cells were washed and re-suspended in binding buffer. Annexin V-FITC (5 μl) was added to samples and incubated for 15 min in the dark, followed by addition of 10 μl PI for 5 min in the dark. Apoptosis was measured by a flow cytometer instrument (Beckman, United States).

### Western Blot

Treated chondrocytes were washed twice in PBS and lysed in lysis buffer (P0013B, Beyotime, China) with 1% proteinase inhibitors. The homogenate was centrifuged at 13200 rpm/min for 30 min to remove insoluble materials, followed by the addition of SDS loading buffer. The total protein concentration was determined with the BCA protein quantification method (Beyotime, China). An equal amount of sample was loaded onto a 10% SDS gel and transferred to polyvinylidene fluoride (PVDF) membrane via electrophoresis. Following blockage with non-fat milk, membranes were incubated with specific anti-β-actin (Bioss, 1:1000), anti-PARP (Cell Signaling Technology, 1:1000), anti-Bcl-2 (Bioss, 1:500), anti-Bax (Cell Signaling Technology, 1:1000), anti-IHH (Abcam, 1:1000) and anti-TRPM7 (Abcam, 1:1000) overnight. Membranes were subsequently washed with Tris buffer saline (TBS) containing 0.24% Tween-20 and incubated with horseradish peroxidase-conjugated secondary antibody for 60 min. The electrochemiluminescence (ECL) reagent was used for visualization of protein signals.

### Quantitative Real-Time PCR

Expression of TRPM7, MMP-13, IL-6 and ADAMTS-5 mRNA was detected with qRT-PCR. Total RNA was extracted from chondrocytes using TRIzol reagent, the concentration determined *via* spectrophotometry at 260 nm and purity assessed based on the 260/280 ratio. Firstly, total RNA was reverse transcribed into complementary DNA (cDNA) and then cDNA was used for qPCR. The expression of relative mRNA was detected under the following conditions: 95°C for 10 min, followed by 40 cycles of 95 °C for 5 s, 61 °C for 10 s, finally by the detection of meltcurve from 65 to 95°C. The primer sequences are listed in Table 1. mRNA levels of GAPDH were used as an endogenous control, and ΔΔCt values were calculated after GAPDH normalization. Fluorescence signals were collected in the extension stage, the Ct value of the sample was recorded, and the transcription level was analyzed by the 2^−ΔΔCt^ method.

**TABLE 1:** Primers of Targeted Genes.

Primer name	Sequence (5′a to 3′a)
GAPDH-F	CTG​CTC​CTC​CTG​TTC​GAC​AGT
GAPDH-R	CCG​TTG​ACT​CCG​ACC​TTC​AC
MMP-13-F	CTG​ACC​TGG​GAT​TTC​CAA​AA
MMP-13-R	ACA​CGT​GGT​TCC​CTG​AGA​AG
TRPM7-F	CTG​AAG​AGG​AAT​GAC​TAC​AC
TRPM7-R	ACA​GGA​AAA​AGA​GAG​GGA​G
ADAMTS-5-F	CGA​CAA​GAG​TCT​GGA​GGT​GAG
ADAMTS-5-R	CGT​GAG​CCA​CAG​TGA​AAG​C
IL-6-F	AGT​CCT​GAT​CCA​GTT​CCT​GC
IL-6-R	CTA​CAT​TTG​CCG​AAG​AGC​CC

### Measurement of Intracellular ROS

Chondrocytes were seeded at a density of 2 × 10^6^ cells/well in 6-well plates and cultured for 24 h, then treated with SNP (0.5 mM) and 2-APB (100 μM) for 12 h. ROS in cells was measured by DCFH-DA fluorescence intensity. The original medium on the culture plate was replaced with DMEM without FBS. After addition of DCFH-DA (1:1,000) (Beyotime, China), the culture plate was incubated for 20 min in the incubator at 37°C and washed three times with DMEM without FBS, and images obtained under a fluorescence microscope. ROS expression was assessed via flow cytometry. FBS-free DMEM was used for dilution of DCFH-DA (1:1000). The chondrocytes were digested with trypsin, and the cell suspension was centrifuged at 1500 rpm for 5 min at 4°C. Then the collected chondrocytes were suspended in diluted DCFH-DA, placed in a cell incubator at 37°C for 20 min. The cell suspension was collected by centrifugation at 4°C and 1500 rpm for 5 min, and then washed three times in DMEM without FBS. Chondrocytes were measured on a flow cytometer instrument (Beckman, United Ststes).

### TRPM7-Small Interfering (si) RNA Transfection Into Chondrocytes

The siRNA sequence targeting TRPM7 and its negative control (NC) were designed and synthesized by Shanghai GenePharma Co., Ltd as follows: 5′-GUC​UUG​CCA​UGA​AAU​ACU​CUU-3' (Si-TRPM7-F), 5′-GAG​UAU​UUC​AUG​GCA​AGA​CUU-3' (Si-TRPM7-R), and the specific sequence of NC is sense: 5′-UUC​UCC​GAA​CGU​GUC​ACG​UTT-3′, antisense: 5′-ACG​UGA​CAC​GUU​CGG​AGA​ATT-3'. Chondrocytes were grown in 6-well plates and transfected at a density of 50–60% cell. For transfection, 10% FBS medium was added to the culture plates. We diluted the siRNA and jetPEI® required for each well in 100 μl of 150 mM NaCl. We used jetPEI® with a volume of 3.2 μl (for 50 nM siRNA) per μg siRNA, mixed the solution, then added the jetPEI® solution to the siRNA solution and mix well, let it stand at room temperature for 30 min. Then the solution was added to the cell plate for 4 h. After replacing with fresh medium, cells were cultured for 24 h, and then treated with SNP (0.5 mM) for 12 h (Bonnet et al., 2013).

### Animals, Grouping and Treatments

Male Sprague-Dawley (SD) rats weighing between 160–180 g were obtained from Jinan Pengyue Experimental Animal Breeding Co., Ltd, China. Under standard laboratory conditions, rats were exposed to water and a light/dark cycle at a temperature of 22 ± 3°C. After adaptation for 7 days, rats were randomly divided into Control (*n* = 8) and AA groups (*n* = 40). The AA group was subdivided into the following groups (*n* = 8): untreated AA, low-dose 2-APB treatment (25 μM), medium-dose 2-APB treatment (50 μM), high-dose 2-APB treatment (100 μM) and positive drug Methotrexate (MTX) treatment. The experimental procedure was authorized by the Anhui Medical University "Experimental animal care and use ethics" committee and conducted in accordance with the US National Institutes of Health "Care and Use of Laboratory Animals Guide". Adjuvant arthritis was induced in rats by injecting 0.1 ml of Complete Freund’s adjuvant (CFA, Chondrex Inc, Redmond WA, United States) at the palmar external region of the right hind paw on day 0. The Control group was injected with the same amount of saline. On day 14 after immunization, 0.1 ml of 2-APB was injected into the joint cavity every three days and MTX (0.5 mg/kg/three days) delivered via intragastric administration until day 26. The control group (day 0) received no drug injection.

### Radiological Examination

Rats were anesthetized with 10% chloral hydrate on day 26 of CFA immunization and subjected to X-rays to detect bone alterations. The hind paw was placed on the photographic film 90 cm from the X-ray light source to obtain a later viewing angle.

### Arthritis Score and Foot Swelling

From the 12th day of the experiment, the arthritis score and foot swelling were assessed every 2 days. The standards of arthritic scoring were evaluated as follows: 0: normal; 1: knuckle swelling; 2: swelling of the ankle or wrist; 3: grievous swelling of the whole paw; 4: foot deformity or stiffness (Chang et al., 2011). The score for arthritis was obtained by two experienced researchers blinded to group allocation. The degree of foot swelling of AA rats was detected using a specific foot swelling instrument (PV-200, Taimeng, China).

### Sample Harvesting and Preparation

Articular cartilage was extracted from the tibial platform of rat for western blot analysis. The ankle joint was set in 4% paraformaldehyde for 24 h at room temperature and decalcified with 10% ethylenediaminetetraacetic acid (EDTA) for 5 weeks for histological and immunohistochemical analyses (Chen et al., 2018).

### Histological Analysis

The prepared ankle joint was dehydrated in high-concentration ethanol, embedded with paraffin and sectioned. The uncalcified sections that had not been dried were used for hematoxylin and eosin (H&E) or safranin O staining immediately.

### Immunohistochemical Analysis

Joint tissue was sequentially dehydrated, embedded, sectioned and dried for IHC analysis. The tissue was deparaffinized, dehydrated, and rinsed with PBS three times for 5 min each time, followed by incubation with 3% H_2_O_2_ for 10 min at 37°C. After rinsing three times as above, tissues were incubated with goat serum blocking solution. Excess liquid was removed, diluted polyclonal primary antibody added dropwise, and sections placed in a humidified cabinet at 4°C overnight. Following equilibration at 37°C for 20 min, sections were rinsed three times as above and incubated with horseradish peroxidase-labeled secondary incubation antibody at 37°C for 20 min. Sections were rinsed as above, yellow DAB (3,3′-diaminobenzidine), tetrahydrochloride, Boster Co., Ltd.) dye applied for 5 min, washed three times with distilled water for 3 min each time, and counterstained with hematoxylin for 30 s. Tissues were rinsed twice with water every 2 min and hydrochloric acid for 30 s, ammonia for 7 min, and distilled water 3 times for 2 min each. Excess liquid was removed, the tissue sealed, and observed under a microscope.

### Statistical Analysis

Statistical comparisons were conducted by using GraphPad Prism 6. All data were expressed as means ± SEM. To compare the means between all measured variables, a one-way analysis of variance (ANOVA) followed by Dunnett test or unpaired Student t test as appropriate. Data were considered significantly different at *P* < 0.05.

## Results

### Effects of Blocking TRPM7 on SNP-Induced Rat Chondrocyte Cytotoxicity

**FIGURE 1:**
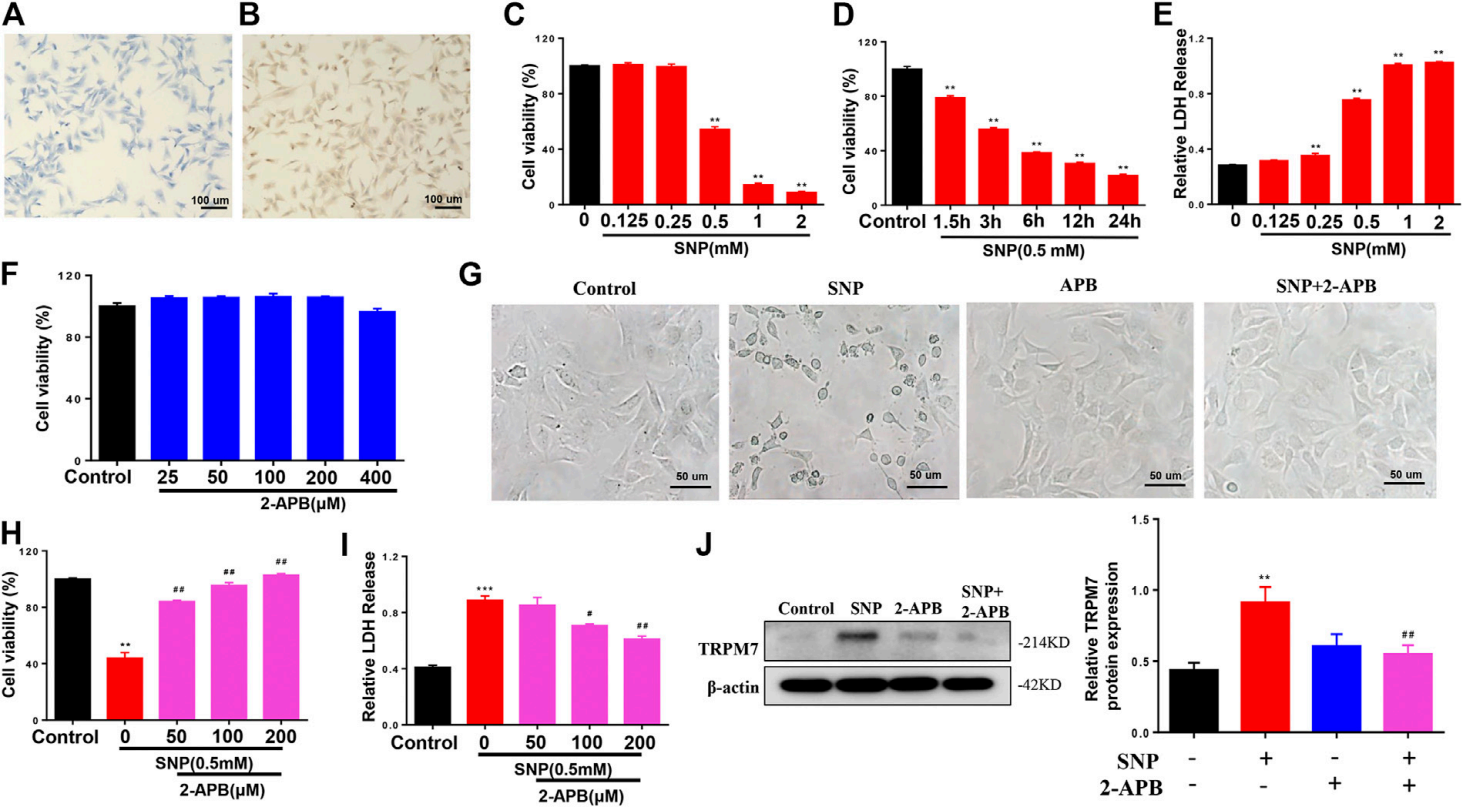
2-APB increases the viability of SNP-treated chondrocytes **(A)** Toluidine blue staining for positive identification of chondrocytes **(B)** Type II collagen staining for chondrocyte identification **(C)** MTT analysis of different concentrations of SNP on chondrocyte viability. Chondrocyte were stimulated by 0.125, 0.25, 0.5, 1.0, 2.0 mM SNP for 12 h (*n* = 6) **(D)** MTT analysis of different treatment time of SNP on chondrocyte viability. Chondrocyte were stimulated by 0.5 mM SNP for 1.5, 3, 6, 12, 24 h (*n* = 6) **(E)** LDH analysis of different concentrations of SNP on chondrocyte toxicity. Chondrocyte were stimulated by 0.125, 0.25, 0.5, 1.0, 2.0 mM SNP for 12 h (*n* = 4) **(F)** MTT analysis of different concentrations of 2-APB on chondrocyte viability. Chondrocyte were stimulated by 25, 50, 100, 200, 400 μM 2-APB for 12 h (*n* = 6) **(G)** Morphology of 2-APB on SNP-treated chondrocytes **(H)** MTT analysis of 2-APB on viability of SNP-treated chondrocytes. Chondrocytes were treated with 0.5 mM SNP and 50, 100, 200 μM 2-APB for 12 h (*n* = 3) **(I)** LDH analysis of 2-APB on chondrocyte toxicity induced by SNP. Chondrocytes were treated with 0.5 mMSNP and 50, 100, 200 μM2-APB for 12 h (n = 4). **(J)** Western blot analysis of 2-APB on SNP-induced TRPM7 protein expression in chondrocytes. Chondrocytes were treated with 0.5 mM SNP and 50, 100, 200 μM 2-APB for 12 h (*n* = 4). Values are presented as means ± SEM. **p < 0.01, ***p < 0.001, compared with control group; ^#^ *p* < 0.05, ^##^ *p* < 0.01, compared with SNP group.

To investigate the effect of TRPM7 on injury in chondrocytes, SNP was used to be as injury inducer, and 2-APB was used to block TRPM7. We initially identified chondrocytes via toluidine blue (light blue) staining (Figure 1A). All cells expressed type II collagen, visualized as a yellow-brown stain (Figure 1B). The cell viability of SNP-induced chondrocytes with or without 2-APB treatment was further examined. Within a certain range, the effect of SNP on cell viability was negatively correlated with concentration and processing time (Figures 1C,D). In addition to cell viability assays, we analyzed SNP-stimulated cell injury by measurement of LDH release. SNP induced a significant increase in LDH release in chondrocytes (Figure 1E). Through the analysis of MTT and LDH, we found that the cell death rate of chondrocytes was about 50% when treated with 0.5 mM SNP for 12 h, which is consistent with previous study (Tonomura et al., 2006). Therefore, chondrocytes were treated with 0.5 mM SNP for 12 h to induce apoptosis. MTT analysis showed that treatment with 2-APB (25–400 μM for 12 h) did not affect cell viability (Figure 1F). Earlier results have demonstrated that TRPM7 exerted significant effects on cell damage. In our experiments, SNP treatment induced shrinkage and death of chondrocytes, which could be effectively inhibited by 2-APB treatment (Figure 1G). As expected, treatment with 2-APB (100 μM, 12 h) reversed the SNP-stimulated decrease in cell viability (Figure 1H). Data from the LDH assay showed that 2-APB decreased the cytotoxicity of SNP on chondrocytes (Figure 1I). SNP induced a significant increase in TRPM7 expression, which could be reversed by 2-APB (Figure 1J), clearly suggesting that the TRPM7 channel plays a significant role in SNP-induced cell damage. Blockage of TRPM7 with 2-APB could therefore effectively reduce SNP-induced cell injury.

### Effects of Blockage or Silence of TRPM7 on Chondrocyte Apoptosis Stimulated by SNP

**FIGURE 2:**
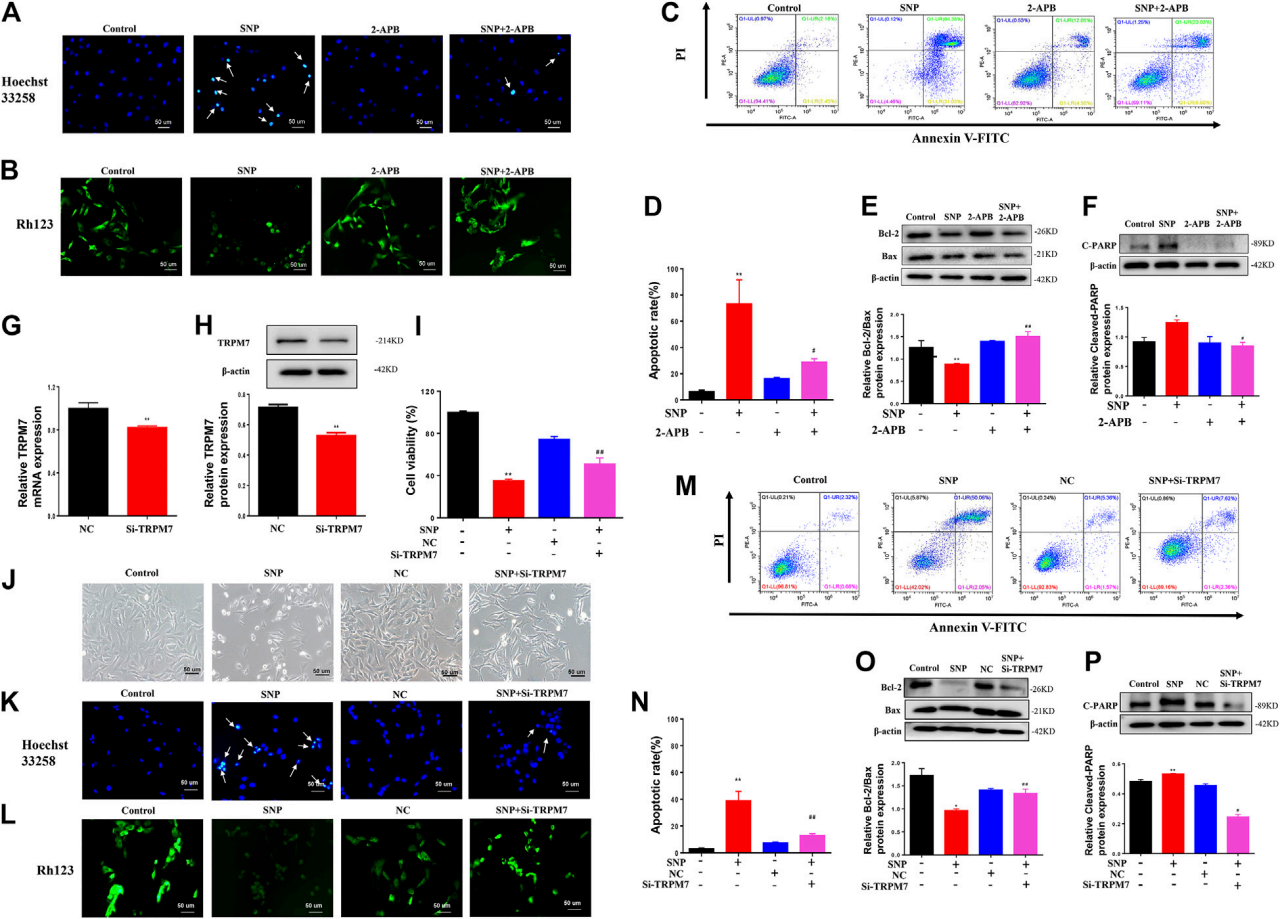
2-APB or Si-TRPM7 reduces SNP-induced chondrocyte apoptosis **(A)** Hoechst 33,258 stain of 2-APB on SNP-induced chondrocyte chromatin of the nucleus (*n* = 3) **(B)** Rh123 satin of 2-APB on SNP-induced mitochondrial membrane potential (*n* = 3) **(C)** Annexin V-FITC and PI satin of 2-APB on SNP-induced chondrocyte apoptosis (*n* = 3) **(D)** Quantitative analysis of chondrocyte apoptosis rate (*n* = 3) **(E)** Western blot analysis of 2-APB on the Bcl-2/Bax ratio (*n* = 3) **(F)** Western blot analysis of 2-APB on cleaved PARP protein expression (*n* = 3) **(G)** qRT-PCR analysis of TRPM7 mRNA expression in chondrocytes treated with Si-TRPM7 (*n* = 3) **(H)** Western blot analysis of TRPM7 protein expression in chondrocytes treated with Si-TRPM7 (*n* = 3) **(I)** MTT analysis of Si-TRPM7 on cell viability of SNP-treated chondrocytes (*n* = 6) **(J)** Morphology of Si-TRPM7 on SNP-induced chondrocytes (*n* = 3) **(K)** Hoechst 33,258 stain of Si-TRPM7 on SNP-induced chondrocyte chromatin of the nucleus (*n* = 3) **(L)** Rh123 satin of Si-TRPM7 on SNP-induced mitochondrial membrane potential (*n* = 3) **(M)** Annexin V-FITC and PI satin of Si-TRPM7 on SNP-induced chondrocyte apoptosis (*n* = 3) **(N)** Quantitative analysis of chondrocyte apoptosis rate (*n* = 3) **(O)** Western blot analysis of Si-TRPM7 on Bcl-2/Bax ratio (*n* = 3) **(P)** Western blot analysis of Si-TRPM7 on cleaved PARP protein expression (*n* = 3). Chondrocytes were seeded in 6-well plates cultured for 24 h, then treated with 2-APB (100 μM) and SNP (0.5 mM) for 12 h, or chondrocytes were seeded in 6-well plates, cultured for 12 h, after transfected with Si-TRPM7 for 4 h and cultured for 24 h, then SNP (0.5 mM) was added and incubated for 12 h. Values are presented as mean ± SEM. ^*^ P < 0.05, ^**^ *p* < 0.01, compared with control group; ^#^ *p* < 0.05, ^##^ *p* < 0.01, compared with SNP group.

Hoechst33258, Rh123 and Annexin V-FITC/propidium iodide (PI) dual staining assays, along with western blot, were employed to detect the effects of blocking or silencing TRPM7 on chondrocyte apoptosis stimulated by SNP. Hoechst33258 staining results showed that chondrocytes treatment with SNP (0.5 mM, 12 h) displayed more nuclear chromatin condensation or fragmentation, which could be suppressed by treatment with 2-APB (Figure 2A). Rh123 staining disclosed that the mitochondrial membrane potential decreased with SNP treatment and was conversely increased in the presence of 2-APB (Figure 2B). In the Annexin V-FITC/propidium iodide (PI) dual staining assay, blocking TRPM7 induced an obvious reduction in apoptosis rate (Figures 2C,D). Western blot revealed a reduction in the Bcl-2/Bax ratio in chondrocytes treated with SNP, which was increased by 2-APB (Figure 2E). The SNP-induced increase in cleaved PARP expression was significantly suppressed by 2-APB (Figure 2F). Expression of TRPM7 in chondrocytes of the Si-TRPM7 treatment group reduced to 80% normal levels, as determined via qRT-PCR (Figure 2G). Expression of TRPM7 in chondrocytes of the Si-TRPM7 treatment group decreased significantly, as determined via western blot (Figure 2H). MTT results showed that SNP induced a decrease in chondrocyte viability, which was restored upon treatment with Si-TRPM7 (Figure 2I). SNP treatment induced chondrocyte shrinkage and death, which was inhibited upon Si-TRPM7 treatment (Figure 2J). Hoechst33258 staining revealed chondrocytes treatment with SNP (0.5 mM, 12 h) displayed more nuclear chromatin condensation or fragmentation, which could be suppressed by treatment with Si-TRPM7 (Figure 2K). In Rh123 staining experiments, SNP induced a decrease in mitochondrial membrane potential, which was significantly increased in chondrocytes treated with Si-TRPM7 (Figure 2L). Annexin V-FITC/propidium iodide (PI) dual staining assay further confirmed that Si-TRPM7 induced a marked reduction in the rate of apoptosis (Figure 2M). Quantitative analysis of the chondrocyte apoptosis rate was further performed (Figure 2N). Western blot results showed that the Bcl-2/Bax ratio was reduced in chondrocytes stimulated with SNP and conversely increased in the presence of Si-TRPM7 (Figure 2O). Furthermore, SNP-induced cleavage of PARP was suppressed by Si-TRPM7 (Figure 2P). Our collective results suggested that blockage or silence of TRPM7 could effectively inhibit chondrocyte apoptosis.

### Effects of TRPM7 Blockage on SNP-Induced ROS Elevation in Chondrocytes

**FIGURE 3:**
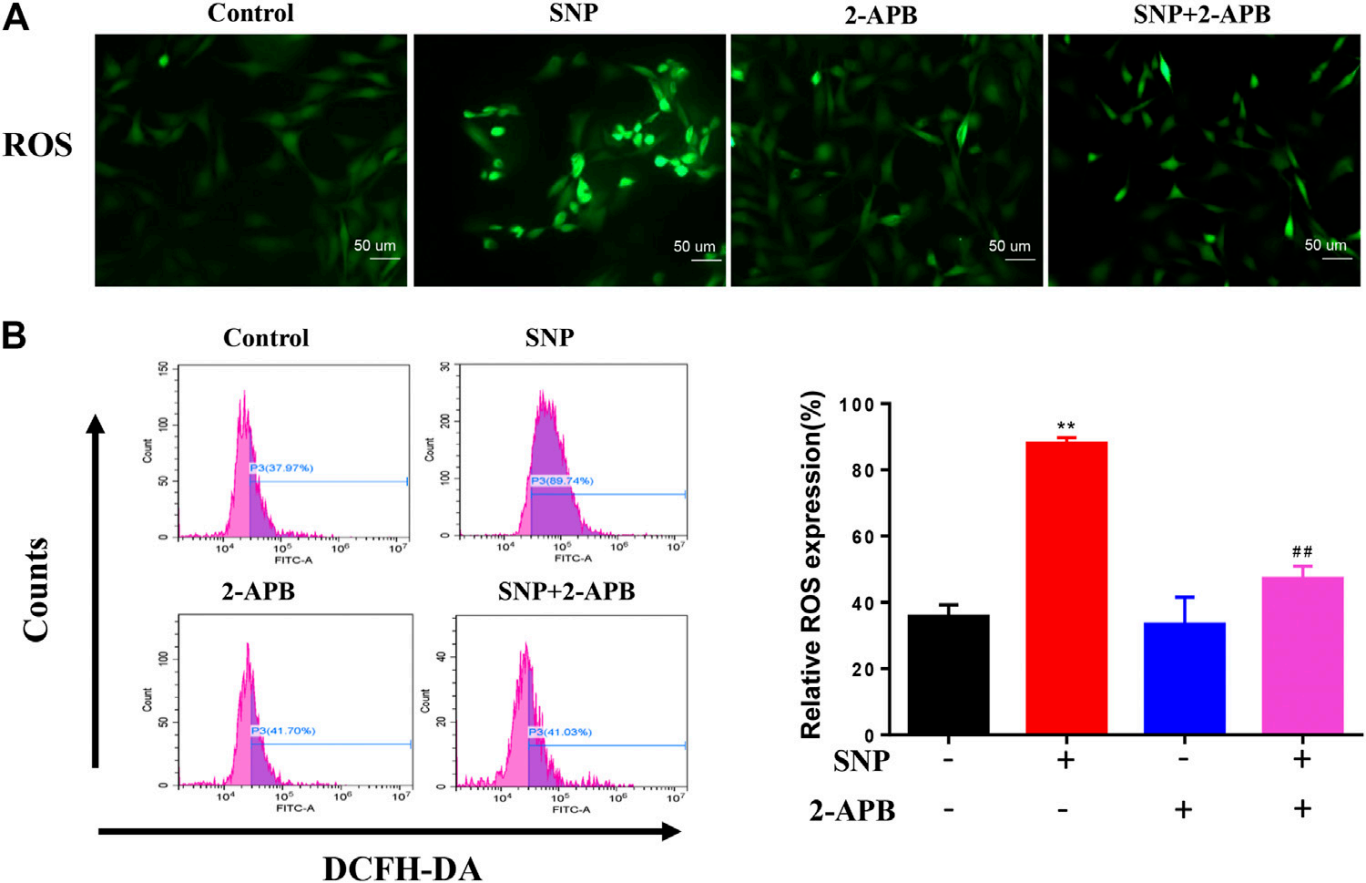
2-APB suppresses SNP-induced ROS expression **(A)** DCFH-DA stain of 2-APB on SNP-induced ROS expression **(B)** Quantitative analysis of 2-APB on SNP-induced ROS expression with flow cytometry. Chondrocytes were seeded in 6-well plates cultured for 24 h, then treated with 2-APB (100 μM) and SNP (0.5 mM) for 12 h. Values are presented as mean ± SEM. *n* = 3, ^**^ *p* < 0.01, compared with control group; ^##^ *p* < 0.01, compared with SNP group.

Crosstalk exists between ROS and Ca^2+^ signaling in mitochondria and Ca^2+^ plays a significant role in maintaining the antioxidant capacity of mitochondria (Bertero and Maack, 2018; Zhou et al., 2019). Since the divalent cation channel TRPM7 transports Ca^2+^, we determined its effects on the production of ROS in mitochondria. To evaluate the effect of 2-APB, ROS production was detected via staining and quantified with flow cytometry. ROS staining showed that SNP treatment led to increased ROS expression, which was significantly decreased in groups treated with 2-APB (Figure 3A). Flow cytometry further validated the marked increase in ROS production in the SNP group (Figure 3B), which was consistently abolished by 2-APB. These findings indicated that generation of ROS induced by SNP is regulated by the TRPM7 channel.

### Effects of Blocking TRPM7 on Expression of Cartilage Procatabolic Proteins

**FIGURE 4:**
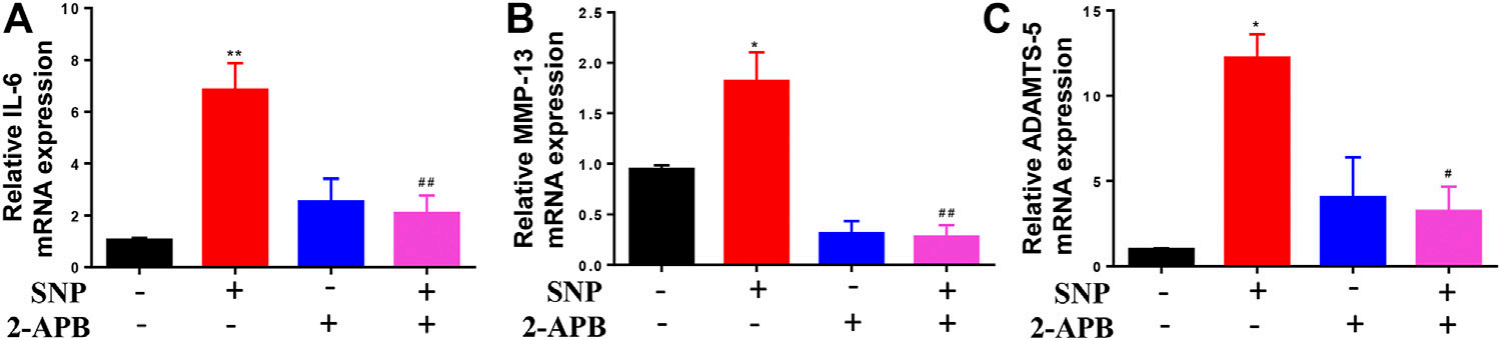
2-APB downregulates IL-6, MMP-13 and ADAMTS-5 mRNA expression in SNP-induced chondrocytes **(A–C)** qRT-PCR analysis of 2-APB on SNP-induced IL-6, MMP-13 and ADAMTS-5 mRNA expression in chondrocytes. Chondrocytes were seeded in 6-well plates cultured for 24 h, then treated with 2-APB (100 μM) and SNP (0.5 mM) for 12 h. Values are presented as mean ± SEM. *n* = 3, ^*^ *p* < 0.05, ^**^ *p* < 0.01, compared with control group; ^#^ *p* < 0.05, ^##^ *p* < 0.01, compared with SNP group.

QRT-PCR was conducted to establish the effects of 2-APB on IL-6, MMP-13 and ADAMTS-5 mRNA levels. Notably, 2-APB treatment suppressed the mRNA levels of IL-6, MMP-13 and ADAMTS-5 upregulated by SNP (Figures 4A–C). Our results indicated that blockage of TRPM7 using 2-APB could reduce SNP-induced extracellular matrix degradation.

### Effects of Activated IHH on Chondrocyte Apoptosis

**FIGURE 5:**
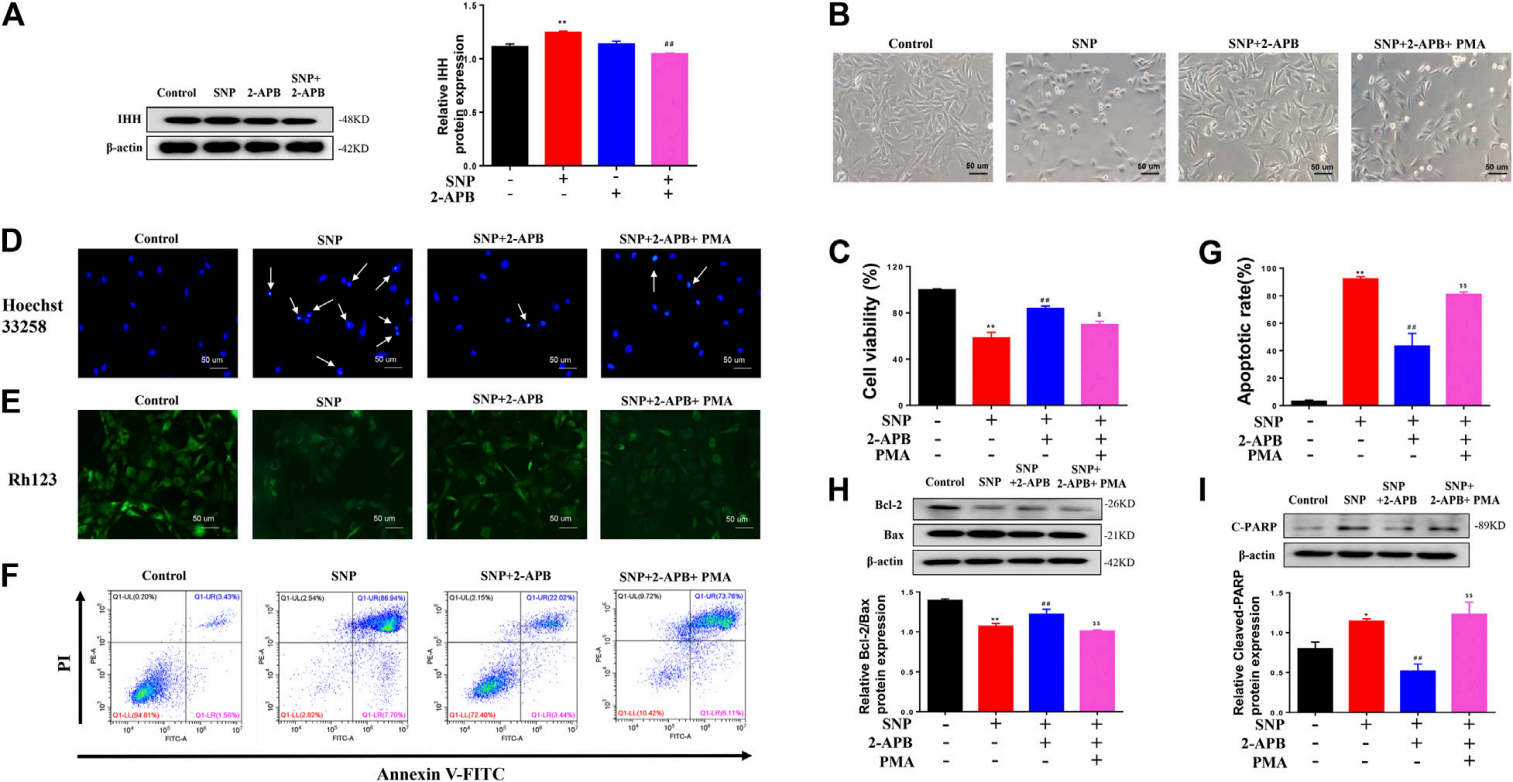
IHH activated by PMA reverses the protective effects of 2-APB on SNP-induced chondrocyte apoptosis **(A)** Western blot analysis of 2-APB on SNP-induced IHH protein expression in chondrocytes (*n* = 3) **(B)** Morphology of PMA on SNP-induced chondrocytes **(C)** MTT assay of PMA on cell viability of SNP-induced chondrocytes (*n* = 6) **(D)** Hoechst 33,258 stain of PMA on SNP-induced chondrocyte chromatin of the nucleus (*n* = 3) **(E)** Rh123 stain of the influence of PMA on mitochondrial membrane potential (*n* = 3) **(F)** Annexin V-FITC and PI statin of SNP-induced chondrocyte apoptosis treated with PMA (*n* = 3) **(G)** Quantitative analysis of chondrocyte apoptosis rate (*n* = 3) **(H)** Western blot analysis of SNP-induced Bcl-2/Bax ratio treated with PMA (*n* = 3) **(I)** Western blot analysis of SNP-induced cleaved PARP protein expression treated with PMA (*n* = 3). Chondrocytes were seeded in 6-well plates cultured for 24 h, then treated with 2-APB (100 μM), SNP (0.5 mM) and PMA (4 μM) for 12 h. Values are presented as mean ± SEM. ^*^ *p* < 0.05, ^**^ *p* < 0.01, compared with the control group; ^##^ *p* < 0.01, compared with the SNP group; ^$^ *p* < 0.05, ^$$^ *p* < 0.01, compared with the SNP+2-APB group.

Clearly, blocking or silencing of TRPM7 reduced apoptosis of chondrocytes triggered by SNP. In view of the finding that IHH is overexpressed in damaged cartilage and regulated by TRPM7 (Zhou et al., 2014; Lu et al., 2017). To explore the role of IHH in TRPM7-mediated chondrocyte apoptosis, purmorphamine (PMA) was used to activate the IHH pathway. Expression of IHH was markedly increased in SNP-induced chondrocytes undergoing apoptosis, which was decreased upon 2-APB treatment (Figure 5A). SNP treatment induced chondrocyte shrinkage and death, which were effectively inhibited after 2-APB treatment. However, treatment with PMA (4 μM) reversed the protective effect of 2-APB on SNP-induced chondrocyte injury (Figure 5B). In the MTT assay, PMA induced a significant reduction in cell viability following combined treatment with SNP and 2-APB (Figure 5C). Hoechst33258 staining results validated that chondrocytes treatment with SNP (0.5 mM, 12 h) displayed more nuclear chromatin condensation or fragmentation, 2-APB (12 h) decreased this phenomenon, which was reversed by PMA (Figure 5D). Rh123 staining further showed that 2-APB enhanced mitochondrial membrane potential reduced by SNP, which was consistently reversed by PMA (Figure 5E). The Annexin V-FITC/propidium iodide (PI) dual staining assay revealed that 2-APB induced a significant decrease in the rate of apoptosis, which was again increased by PMA (Figure 5F). Quantitative analysis of the chondrocyte apoptosis rate was additionally conducted (Figure 5G). In western blots, the Bcl-2/Bax ratio was reduced in chondrocytes treated with SNP, which was increased by 2-APB and subsequently decreased upon PMA treatment (Figure 5H). SNP-induced cleavage of PARP was suppressed following treatment with 2-APB. However, expression of cleaved PARP was increased after PMA treatment (Figure 5I). Based on these findings, we proposed that SNP-induced apoptosis is regulated by TRPM7-mediated IHH activation.

### Effects of 2-APB on Clinical Signs in AA Rats

**FIGURE 6:**
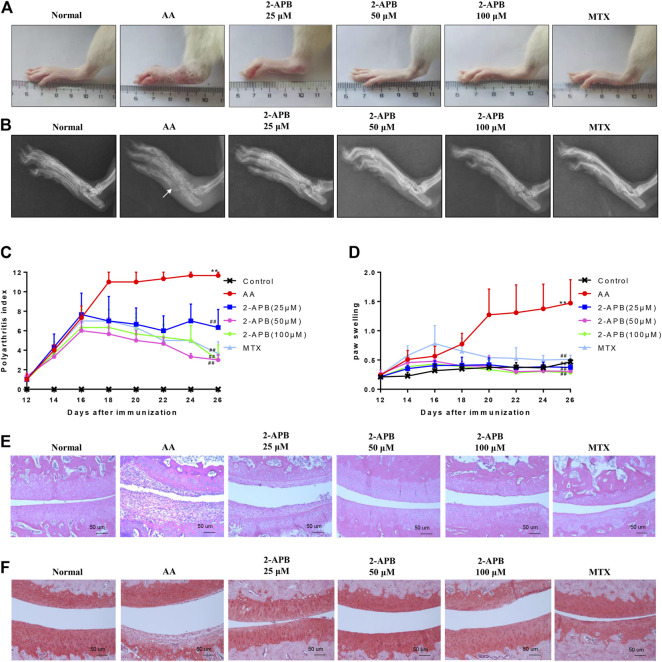
2-APB attenuates the clinical indications of ankle joint damage in AA rats **(A)** Representative images of rat paws from each group **(B)** Representative radiological images of rat paws from each group. White arrow: bone erosion **(C,D)** On days 12–26 after immunization, arthritis score and foot swelling of SD rats were recorded and assessed every two days **(E)** HE staining of ankle joint in rats from each group **(F)** Safranin O staining of ankle joint in rats from each group. Values are presented as mean ± SEM. *n* = 8, ^**^ *p* < 0.01, compared with the control group; ^##^ *p* < 0.01, compared with the AA group.

To further demonstrate the influence of 2-APB on adjuvant arthritis, experiments were performed on AA rats injected with CFA. 2-APB reduced the clinical indications of ankle joint damage in AA rats in a dose-dependent manner (Figure 6A). Imaging results revealed bone erosion in the AA group and an increased gap between bones, compared with the control group. Treatment with 2-APB reduced bone erosion and bone gap to a significant extent, compared to the AA group (Figure 6B). On days 12–16 after immunization, paw swelling and arthritis scores of the AA group were markedly increased relative to the control group. On days 18–26 after immunization, ankle joint and arthritis scores of MTX and 2-APB groups were significantly reduced relative to the AA group. The lower concentration of 2-APB (25 μM) exerted a slight protective effect on arthritis score and paw swelling while 50 and 100 μM 2-APB had significant protective effects (Figures 6C,D). The results of HE staining showed that articular cartilage erosion and synovial hyperplasia were significantly increased in the AA group, which were inhibited by 2-APB (Figure 6E). Safranin O stain showed that compared with the control group, articular cartilage damage in the AA group was severe and could be reversed by 2-APB (Figure 6F). These results suggested that blocking of TRPM7 with 2-APB attenuates the clinical indications of ankle joint damage in AA rats.

### Effects of 2-APB on Cartilage Degradation and Apoptotic Proteins in Articular Cartilage of AA Rats

**FIGURE 7:**
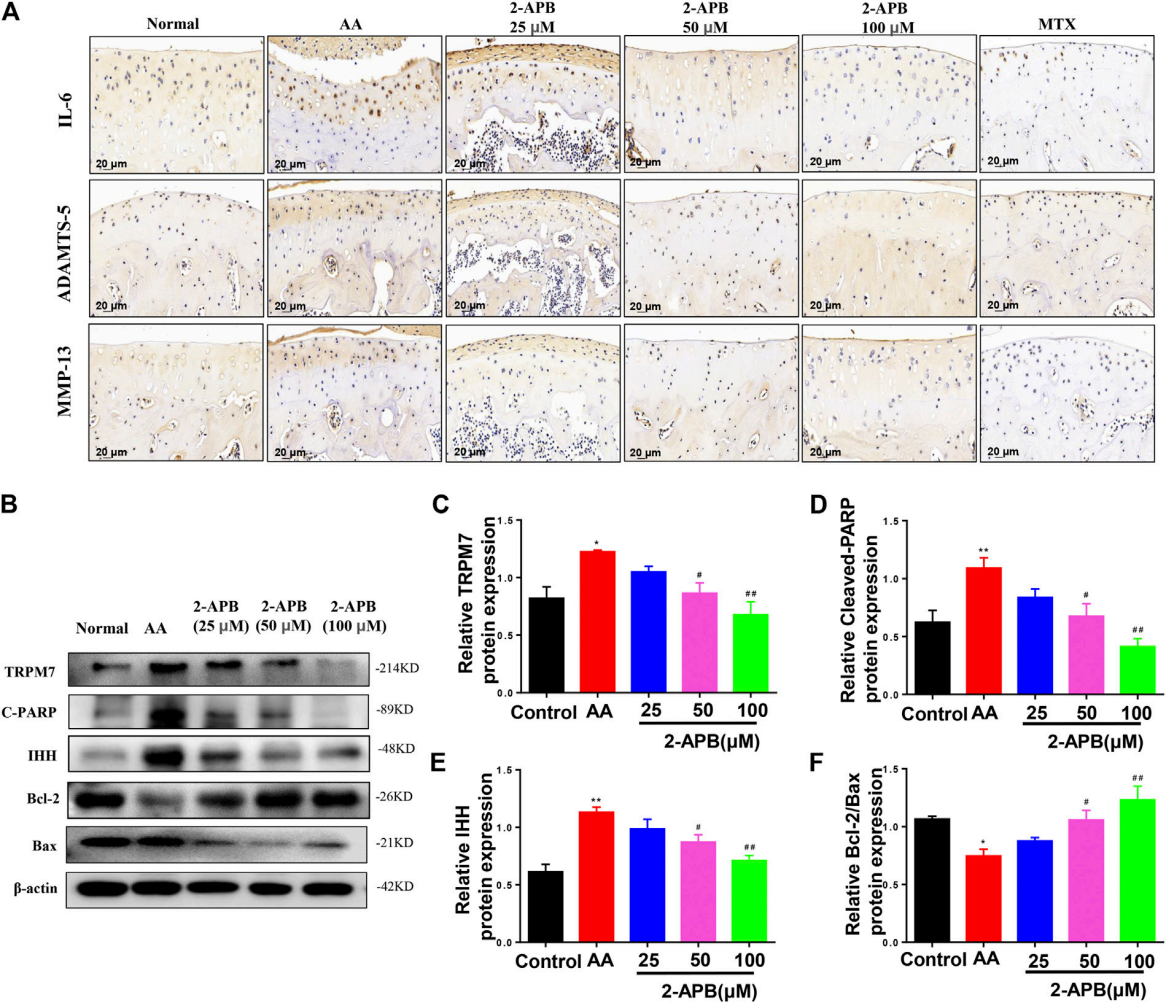
2-APB suppresses IL-6, ADAMTS-5 and MMP-13 protein expression in AA rat articular cartilage through inhibiting IHH expression **(A)** Immunohistochemical analysis of 2-APB on expression of IL-6, ADAMTS-5 and MMP-13 in AA rats **(B–F)** Western blot analysis of 2-APB regulation of TRPM7, IHH and cleaved PARP expression and Bcl-2/Bax ratio in articular cartilage of AA rats. Values are presented as mean ± SEM. *n* = 8, ^*^ *p* < 0.05, ^**^ *p* < 0.01, compared with control group; ^*#*^ *p* < 0.05, ^##^ *p* < 0.01, compared with AA group.

Results from immunohistochemical experiments showed increased IL-6, MMP-13, and ADAMTS-5 levels in articular cartilage of AA rats that were suppressed by following 2-APB treatment (Figure7A). Western blot results showed increased expression of cleaved PARP, IHH and TRPM7 and decreased Bcl-2/Bax ratio in the AA group relative to the control group. Consistently, 2-APB induced a decrease in TRPM7, IHH and cleaved PARP expression and increase in Bcl-2/Bax ratio (Figures 7B–F). Our results support a critical role of TRPM7 in cartilage degradation and apoptosis.

## Discussion

In this study, we explored the influence of 2-APB on articular cartilage damage in an adjuvant-induced arthritis rat model and SNP-induced chondrocyte apoptosis, along with potential associations and mechanisms of action of TRPM7 and IHH. SNP exerted no significant effects on chondrocyte viability at a concentration of 0.25 mM. However, at 0.5 mM SNP, even following treatment for 1.5 h, chondrocyte viability was significantly decreased. Concomitantly, SNP induced a marked increase in expression of TRPM7. 2-APB significantly reversed the decline in chondrocyte viability triggered by SNP and reduced TRPM7 expression. Our results indicate that 2-APB suppresses chondrocyte viability through the TRPM7 channel. Data from *in vivo* experiments showed that apoptosis occurring in articular cartilage cells and cartilage damage of the AA model could be reversed upon blockage of TRPM7.

TRPM protein is a member of a group of ion channels with variable permeability, expression patterns, physiological functions and activation mechanisms (Cook et al., 2009). Among these, the TRPM7 channel has received widespread attention owing to its high permeability to cations, such as Mg^2+^, Ca^2+^, Zn^2+^. In pheochromocytoma cells, TRPM7 promotes production of ROS, which exert significant effects on cell survival and proliferation (Yang et al., 2016). Earlier studies have shown that embryonic stem cells and malignant B-lymphocytes stagnate in the absence of TRPM7, but are activated and enter the cell cycle upon addition of Mg^2+^ to the medium (Schmitz et al., 2003; Sahni et al., 2010). More recent evidence has confirmed that activation of TRPM7 promotes apoptosis (Malemud, 2017). However, limited reports to date have focused on the biological role of TRPM7 in articular cartilage. We initially proposed that TRPM7 functions in rat articular chondrocyte apoptosis. In the current study, expression of TRPM7 was significantly increased after SNP stimulation in chondrocytes, and treatment with 2-APB could inhibit TRPM7 and attenuate chondrocyte apoptosis stimulated with SNP. Western Blot and flow cytometry analyses illustrated that 2-APB reverses chondrocyte apoptosis stimulated by SNP, upregulates the Bcl-2/Bax ratio and downregulates cleaved PARP expression in cultured chondrocytes. 2-APB is not a specific inhibitor of TRPM7 channels, as it affects many ion channels including Orai/CRACM channels. To further confirm the role of TRPM7 in chondrocyte apoptosis, we also conducted parallel verification by silencing TRPM7. Silencing of TRPM7 induced a significant increase in cell viability and Bcl-2/Bax ratio along with concomitant reduction of cleaved PARP expression. Our flow cytometry results showed that 2-APB significantly reduces SNP-induced early apoptosis compared with the SNP group. 2-APB could also reduce SNP-induced late apoptosis from ∼60 to ∼20%, and may not be able to completely inhibit late apoptosis, which is consistent with the research (Liang et al., 2018). This may also indicate that TRPM7 may play a key role in the initiation of apoptosis. Therefore, it is necessary to further explore the specific mechanism of TRPM7 regulating early and late apoptosis in the future. Our collective results clearly demonstrate that 2-APB inhibits TRPM7 and attenuates chondrocyte apoptosis induced by SNP.

HH, a signaling pathway with a significant effect on embryonic development, is transmitted from the cell membrane to nucleus (Varjosalo and Taipale, 2008). IHH belonging to the HH family participates in endochondral bone formation (Skoda et al., 2018). Earlier studies have shown that IHH is related to development of the breast and gastrointestinal tract (van den Brink et al., 2004). As a unique mechanism of associated liver partition and portal vein ligation for staged hepatectomy, IHH accelerates liver regeneration (Langiewicz et al., 2018). IHH exerts significant effects on chondrocyte hypertrophy and proliferation (Zhang et al., 2018b). However, its specific function in chondrocyte apoptosis remains unclear at present. In our experiments, IHH expression was significantly increased after SNP treatment and reduced by 2-APB. Notably, 2-APB suppressed SNP-induced chondrocyte apoptosis, while PMA (an IHH activator) reversed the increased cell viability induced by 2-APB, downregulated the Bcl-2/Bax ratio and upregulated cleaved PARP protein expression. The study has shown that TRPM7 may be related to IHH in hypertrophic chondrocyte, and this association is mediated by Ca^2+^ (Lu et al., 2017). Our results showed that the expression of TRPM7 and IHH increased significantly in SNP-treated chondrocytes, while the expression of IHH was significantly decreased after blocking TRPM7. We also found that blocking TRPM7 could reduce SNP-induced chondrocyte apoptosis, while activation of IHH using PMA could reverse this protective effect. These results support that TRPM7 modulates apoptosis by regulating IHH. However, the specific mechanism of TRPM7 regulating IHH is still unclear, which still needs future research to explore.

**FIGURE 8:**
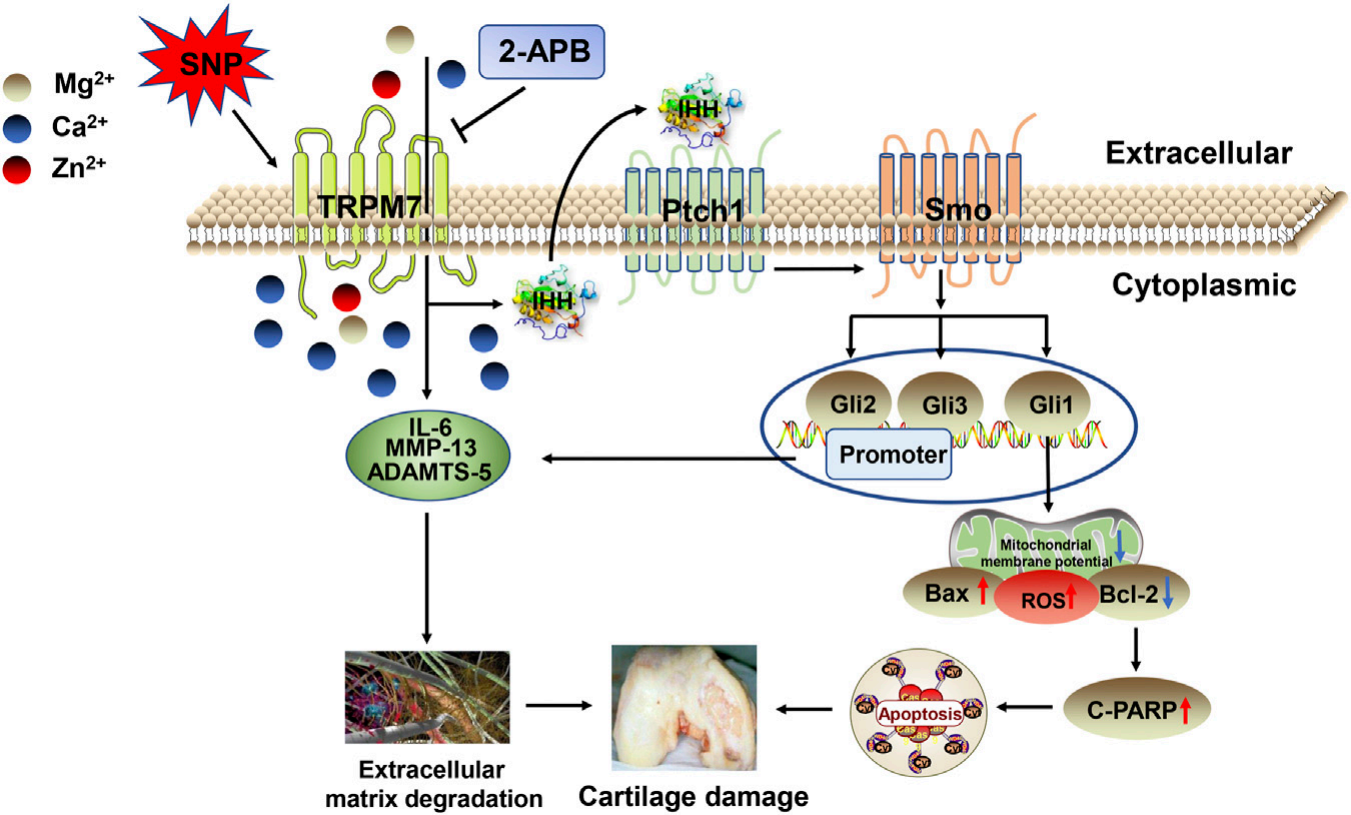
Schematic depicting the roles of TRPM7 and IHH in articular chondrocyte apoptosis. SNP triggers an increase in TRPM7 and IHH and promotes the expression of apoptotic proteins, leading to chondrocyte apoptosis. Activation of TRPM7 and IHH also enhances expression of IL-6, MMP-13, and ADAMTS-5, which promotes cartilage damage and aggravates the process of arthritis.

The predominant mechanism of cartilage damage in arthritis is chondrocyte apoptosis (Zhou et al., 2015a; Zhou et al., 2018b). Suppression of apoptosis can therefore improve the development of articular cartilage (Zhou et al., 2015b). Consistently, data from our *in vivo* experiments showed a significant increase in TRPM7 and IHH expression in articular cartilage of AA rats. Moreover, expression of cleaved PARP in cartilage was obviously increased and Bcl-2/Bax ratio decreased, eventually resulting in AA rat cartilage damage. These findings indicate that chondrocyte apoptosis, which is regulated by the TRPM7/IHH pathway, potentially contributes to AA rat cartilage injury. In addition, low-dose 2-APB injection into the joint cavity had some obvious therapeutic effect, and medium and high doses significantly reversed articular cartilage damage in AA rats. Taken together, the results indicate that inhibition of the TRPM7/IHH axis exerts a protective effect against cartilage damage in AA rats. MMPs, in particular, MMP-13, decompose proteoglycans in the extracellular matrix. Blockage of TRPM7 with 2-APB could effectively inhibit MMP-13 expression, reduce cartilage damage and alleviate disease (Figure 8).

In summary, our study has illustrated for the first time that blockade of TRPM7 inhibits SNP-induced apoptosis of articular chondrocytes and reduces cartilage damage in AA rats. 2-APB contributes to cartilage protection *in vivo* by inhibiting the TRPM7 channel and IHH signaling and supports its potential therapeutic value in treatment of arthritis with articular cartilage damage.

## Conclusion

In conclusion, TRPM7 is highly expressed in chondrocytes and articular cartilage in AA rats and its blockade alleviates chondrocyte apoptosis and articular cartilage damage in AA rats through modulation of the IHH signaling pathway. Our collective findings support an important role of TRPM7 in chondrocyte apoptosis and its potential utility as a therapeutic target for the arthritis with articular cartilage damage.

## Data Availability

The raw data supporting the conclusions of this article will be made available by the authors, without undue reservation.
